# Facial Adipostructuring With Biochemical Modulation in Chronic Peripheral Facial Paralysis: A Case Report

**DOI:** 10.7759/cureus.111350

**Published:** 2026-06-23

**Authors:** Hector Calfin, Veronica Riquelme, Gladys Velazco, Manuel Sequera, César Castro

**Affiliations:** 1 Aesthetic Medicine, Climedent Puerto Montt, Puerto Montt, CHL; 2 Rehabilitation Sciences and Quality of Life, Universidad San Sebastian, Puerto Montt, CHL; 3 Facial Harmonization, Centro Latinoamericano de Entrenamiento Médico e investigación, Bogota, COL; 4 Medicine and Anatomy, Universidad de Los Andes, Mérida, VEN; 5 Medical Academy, Academia Icrom Medical, Santiago, CHL; 6 Dentistry, Universidad San Sebastian, Puerto Montt, CHL

**Keywords:** chronic peripheral facial paralysis, dermal white adipose tissue, dimethylaminoethanol, facial adipostructuring, neuromuscular rehabilitation, regenerative aesthetic medicine

## Abstract

Chronic peripheral facial paralysis (CPFP) implies a prolonged course without recovery of neuromuscular activity of facial expression and the biological impossibility of restoring spontaneous function. Facial paralyses, most commonly idiopathic in origin, are graded using the House-Brackmann (HB) scale, where grade I represents normal function and grades II through VI indicate progressively greater functional compromise. Furthermore, CPFP lacks satisfactory therapeutic options, and conventional strategies, botulinum toxin and surgical reanimation, neither address the collapse of facial structural support nor the dysfunction of dermal white adipose tissue (dWAT) that accompanies chronic denervation. This report presents a treatment strategy for patients with CPFP consisting of Facial Adipostructuring (AEF), a biomechanical reorganization of fat compartments via cannula-based mechanotransduction without tissue extraction, combined with biochemical modulation using dimethylaminoethanol (DMAE), organic silicon, and acetyl hexapeptide-38. The patient progressed from HB grade IV to grade I over three biweekly sessions, with improvement sustained at nine-month follow-up without additional interventions. The changes observed were consistent with improvements on the HB functional scale and were documented through standardized clinical photography. This treatment represents a minimally invasive regenerative approach that may act on the neuro-muscular-adipose unit through dWAT mechanotransduction and neuromuscular junction modulation. We present the case of a female patient with idiopathic facial paralysis of six years' duration, classified as grade IV on the HB scale, who was treated with the described technique.

## Introduction

Chronic peripheral facial paralysis (CPFP) represents one of the most devastating conditions in neuromusculoskeletal medicine. By affecting the seventh cranial nerve, it abolishes essential functions such as ocular protection, oral competence, and speech articulation, and disrupts the primary vehicle of human nonverbal communication: mimetic expression. When spontaneous recovery mechanisms fail, patients face permanent sequelae including synkinesis, spasms, and facial asymmetry that profoundly impair quality of life [1].

Standard therapeutic options include complex surgical reanimation and palliative conservative management, with botulinum toxin considered a primary treatment for synkinesis control. However, this approach presents a therapeutic paradox; to improve symmetry, it induces chemical paralysis on the unaffected side. Prolonged use requires periodic injections to sustain its effect and does not address the loss of facial structural support that accompanies denervation [1,2].

Atrophy of deep fat compartments and laxity of the facial ligamentous system contribute significantly to ptosis and functional incompetence in the denervated patient [3,4]. Dermal white adipose tissue (dWAT), recently recognized as a dynamic component of the facial neuromuscular unit, shares lineage with dermal fibroblasts and may dedifferentiate into myofibroblasts under pathological conditions, perpetuating fibrosis and blocking functional recovery [5].

We describe a case of facial adipostructuring [6,7] combined with biochemical modulation using dimethylaminoethanol (DMAE) and senolytic agents, organic silicon (OS), hexapeptide-38 (H-38), and centella asiatica (CA), in a patient with idiopathic CPFP of six years’ duration. The objective is to propose a pathophysiological framework for the mechanism of action of this treatment, to document the clinical results, and to contribute to the existing literature on regenerative approaches in CPFP neurorehabilitation.

## Case presentation

History and diagnosis

A 59-year-old female patient with no relevant medical history or chronic conditions presented with three episodes of right-sided idiopathic facial paralysis (Bell’s palsy) over a 10-year period. The patient had no comorbidities, coagulation disorders, active skin lesions in the facial area, or concurrent pharmacological treatment that could interfere with adipose tissue function. The last episode, occurring six years prior to consultation, showed no improvement, establishing a diagnosis of CPFP. During the preceding six years, the patient had undergone neurological evaluation, facial kinesiotherapy, neuromuscular electrical stimulation, and botulinum toxin injections on the unaffected side, without achieving significant functional improvement throughout the entire follow-up period. The patient’s consultation at our clinic was not initially motivated by a request for treatment of her facial paralysis, but rather by an aesthetic request for facial adipostructuring. Consequently, no formal neurological examination, imaging studies, or standardized baseline functional assessments beyond House-Brackmann (HB) classification [8] were performed at our clinic, as the intervention was not initially designed as a therapeutic approach for the paralysis itself.

Clinical examination revealed marked right hemifacial asymmetry, myospasms and blepharospasm, facial muscle pain and fatigue, and functional impairment in smiling, feeding, speech, and facial gesturing. HB classification was grade IV.

Intervention

Facial adipostructuring treatment was implemented over three biweekly sessions (approximately 60 minutes per session) under aseptic conditions, using standardized cannula-based mechanotransduction with a 22 G × 40 mm cannula in fat compartments (three to five anteroposterior vectorial movements per compartment) and a 27 G × 40 mm cannula in interseptal spaces (retroinjection technique without movement). Biochemical modulation was divided into stages according to the function of the bioactive components used, with each bioactive solution administered via the commercially formulated ADIPOESTRUCTUR kit (Mioface Harmony, Bogota, Colombia), at a standardized dose of 2.5 mL per vial per session. Stage 1 (Modeling Solution) contained L-carnitine, caffeine, troxerutine, Melilotus officinalis extract, Centella asiatica, and acetyl hexapeptide-38; Stage 2 (Firming Solution) contained pyruvic acid, caffeine, and other senolytics; Stage 3 (Renewing Solution) contained L-carnitine, methylsilanol mannuronate, sorbic acid, and pyruvic acid; and a specific interseptal solution for the interseptal spaces containing DMAE tartrate and acetyl hexapeptide-38. All products used in each session held certification from the Chilean Public Health Institute (Instituto de Salud Pública de Chile, ISP).

Outcome assessment

Outcomes were assessed using the HB scale at each session and during follow-up at nine months after the last intervention, complemented by standardized clinical photographs at each session and mirror-image photographic analysis for asymmetry quantification. Additional assessments included clinical evaluation by the treating physician (resting symmetry, range of motion during smiling and eyebrow elevation, blepharospasm, and synkinesis) and the patient’s verbal report regarding pain, functionality, and quality of life. From the patient’s perspective, she verbally reported progressive improvement in facial functionality across the treatment sessions. By the nine-month follow-up, she described complete resolution of facial muscle pain and fatigue, recovery of oral competence and facial expression capacity, and a notable improvement in her sense of personal security and self-perception. In her own words, she reported feeling “more secure, free of pain, and more beautiful.” She expressed high overall satisfaction with the treatment outcomes and voluntarily agreed to maintain long-term follow-up visits.

CPFP progression was evaluated according to HB scale grade, improving from IV to I following three biweekly sessions, with sustained maintenance at five months and confirmed at the nine-month follow-up (without additional interventions). This represents a clinically significant and durably sustained improvement. No complications or adverse effects were observed throughout the entire follow-up period.

**Figure 1:**
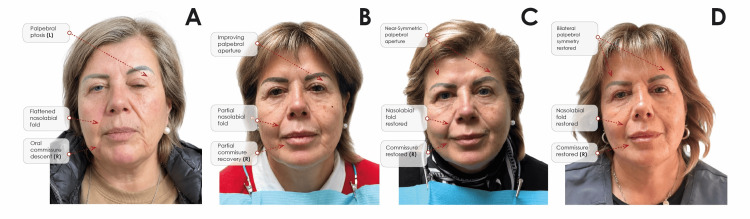
Progressive clinical improvement following facial adipostructuring treatment in chronic peripheral facial paralysis (A) Baseline image (Session 1, House-Brackmann (HB) [8] grade IV); (B) Post Session 2 (15 days after Session 1; HB grade III); (C) Post Session 3 (15 days after Session 2, HB grade I); (D) Five months after Session 1 (HB grade I maintained). Note the progressive recovery of facial symmetry and palpebral aperture. Informed consent was provided by the patient to publish her full facial images without anonymity in this open-access journal.

Serial clinical photographs are shown in Figure 1.

Mirror-image photographic analysis

**Figure 2:**
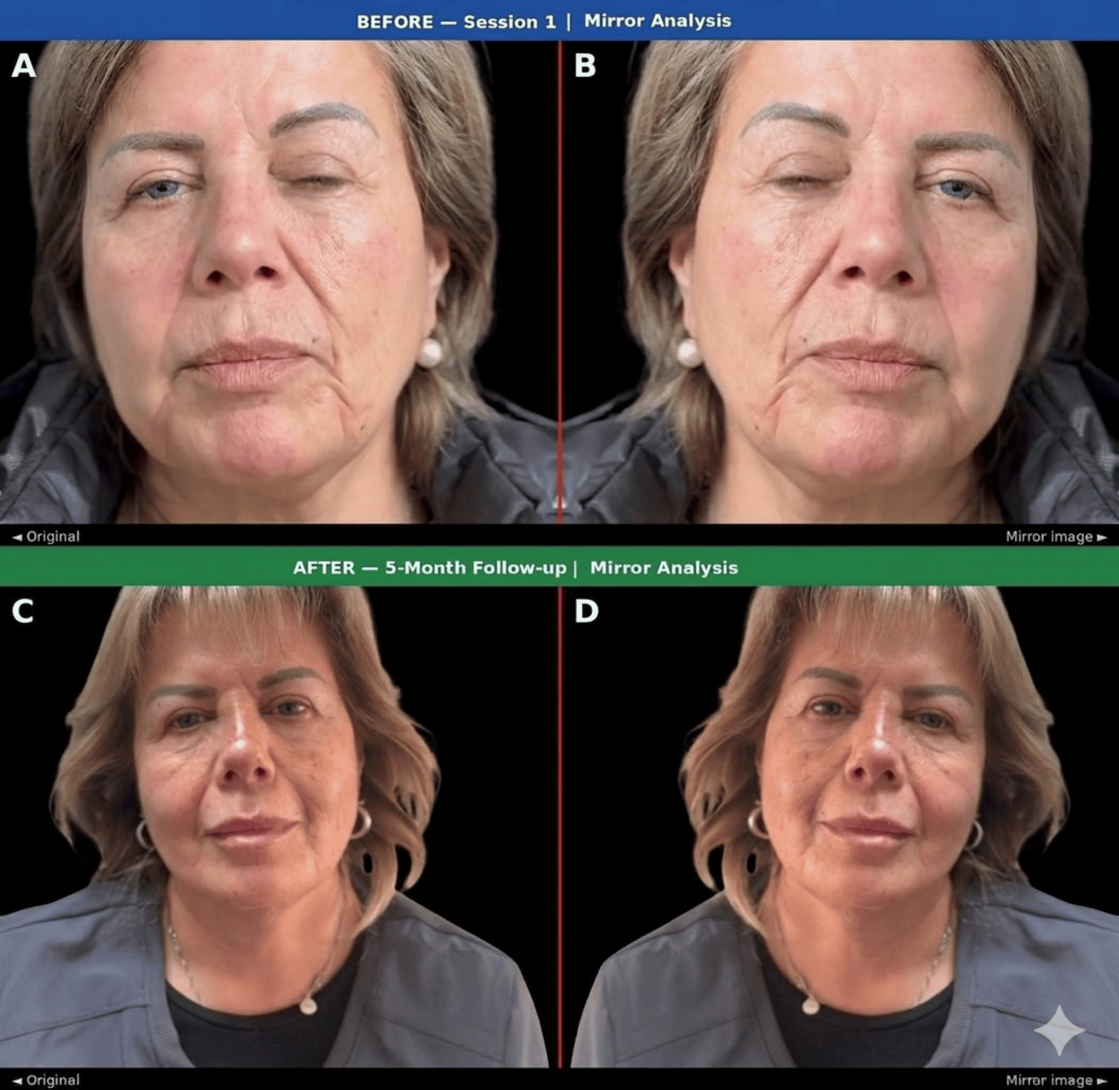
Comparative mirror-image analysis A-B: baseline (HB grade IV); C-D: five-month follow-up (HB grade I). Each image shows the original side (left) and its specular reflection (right), separated by a central red line. The mirrored image is recognizable as the patient’s face, demonstrating the substantial reduction in hemifacial asymmetry. Informed consent was provided by the patient to publish her full facial images without anonymity in this open-access journal.

**Figure 3:**
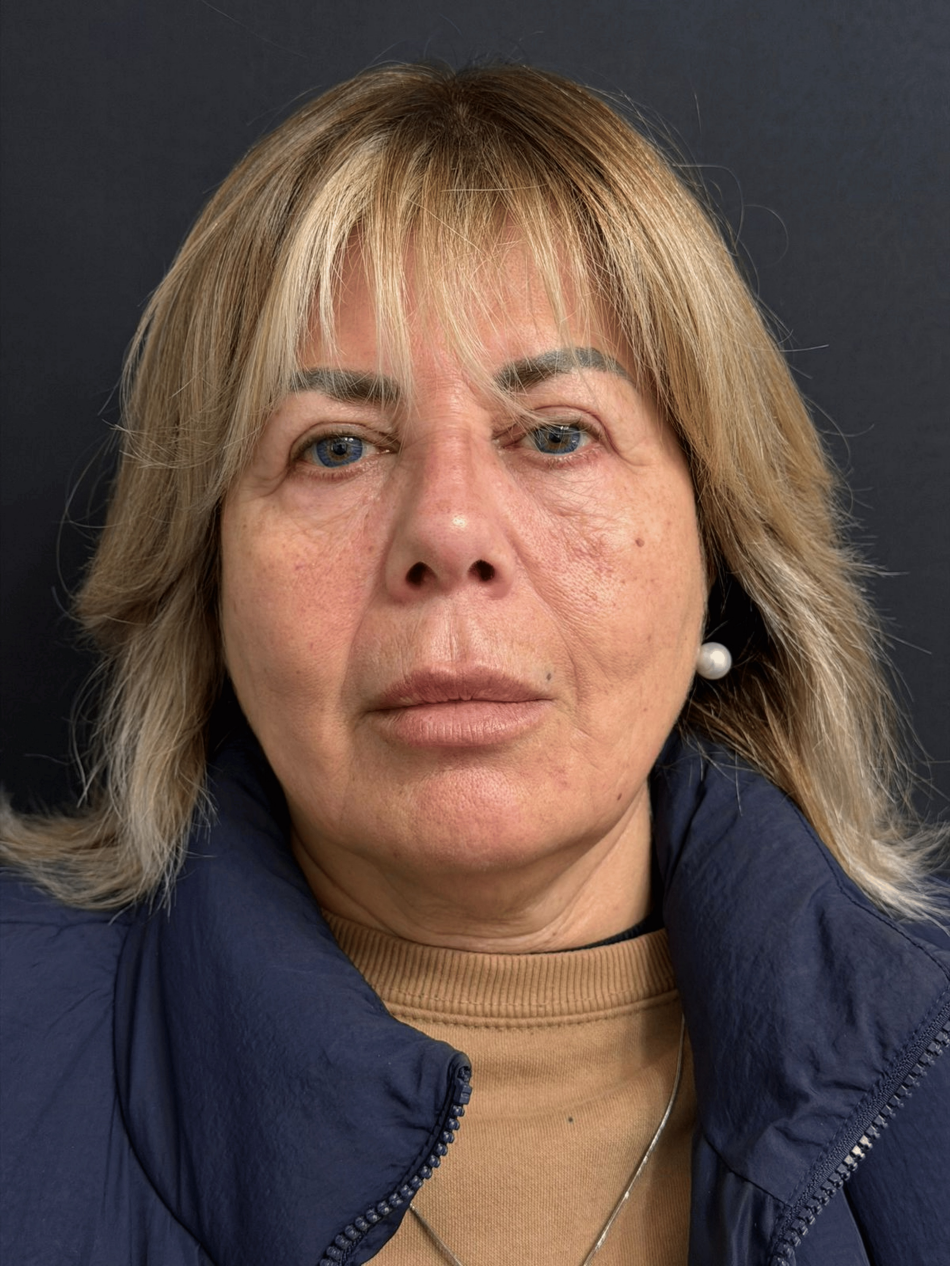
Nine-month follow-up clinical photograph Maintained facial symmetry and palpebral aperture are observed. Informed consent was provided by the patient to publish her full facial images without anonymity in this open-access journal.

Comparative mirror-image analysis (Figure 2) revealed at baseline: marked right upper eyelid ptosis, deflation of the suborbicularis oculi fat (SOOF) malar compartment, deepening of the nasolabial fold, and descent of the oral commissure 5-8 mm relative to the contralateral side. At five months: bilateral equalization of palpebral aperture, restoration of malar projection, attenuation of the nasolabial fold, and repositioning of the oral commissure. The nine-month photographic follow-up (Figure 3) confirms the preservation of these findings, with maintained facial symmetry, bilateral equalization of palpebral aperture, and absence of blepharospasm without additional interventions.

Summary of results by session

The active compounds used across all three sessions, their primary biological functions, and proposed mechanisms of action are summarized in Table 1.

**Table 1:** Active ingredients per session, primary biological function, and proposed mechanism of action with bibliographic support FAS: facial adipostructuring; cAMP: cyclic adenosine monophosphate; CPFP: chronic peripheral facial paralysis; dWAT: dermal white adipose tissue; FFA: free fatty acids; SASP: Senescence-Associated Secretory Phenotype; SOOF: suborbicularis oculi fat; TGF-β: transforming growth factor beta; HB: House-Brackmann; DMAE: dimethylaminoethanol

Session	Ingredient	Biological Function	Proposed Mechanism of Action	References
Session 1 (Day 0) | Modeling Solution + Interseptum (HB IV → III)				
S1	DMAE tartrate	Cholinergic neuromuscular modulation	Motor endplate membrane stabilization; acetylcholine precursor; reduction of ectopic hyperexcitability (synkinesis, blepharospasm)	[9,10]
S1	Acetyl Hexapeptide-38	Localized adipogenesis; restoration of fat volume	Promotion of adipogenesis in SOOF and malar compartments; recovery of hemifacial structural support	[11]
S1	L-carnitine	Lipid metabolism; localized lipolysis	Fatty acid transport to mitochondria; modulation of inflammatory microenvironment via FFA release after mechanotransduction	[4]
S1	Caffeine	Phosphodiesterase inhibition; lipolytic activation	Increased intracellular cAMP in adipocytes; enhanced lipolysis and reorganization of fibrotic compartments	—
S1	Troxerutine	Vascular protection; anti-edema	Reduced capillary permeability and interstitial edema post-mechanotransduction; local antioxidant effect	—
S1	Centella asiatica	Neuroprotector; senolytic; nerve regeneration	Neuritogenesis stimulation; SASP attenuation; fibrosis inhibition via TGF-β regulation	[12,13]
S1	Melilotus officinalis ext.	Lymphatic drainage; anti-inflammatory	Post-intervention edema reduction; facilitation of drainage of the hemifacial fibrotic microenvironment	—
Session 2 (Day 15) | Firming Solution (Progressive Dose) (HB III → II)				
S2	DMAE tartrate (progressive dose)	Progressive optimization of neuromuscular transmission	Gradual increase in residual muscle tone; reinforcement of voluntary motor control without suppressing neuromotor signal	[9,10]
S2	Pyruvic acid	Senolytic; senescent cell remodeling	Selective elimination of senescent cells from the fibrotic microenvironment; SASP reduction; facilitation of tissue regeneration	[14-16]
S2	Caffeine (Stage 2)	Sustained lipolytic activation; synergism with pyruvate	Enhanced reorganization of interseptal fat compartments; synergy with pyruvic acid in eliminating chronic inflammatory microenvironment	—
Session 3 (Day 32) | Renewing Solution (Maintenance) (HB II → I)				
S3	Methylsilanol Mannuronate (Organic Silicon)	Extracellular matrix organization; collagen synthesis	Cross-linking of collagen types I and III in interseptal septa; reinforcement of retaining ligaments; stabilization of reconformed structural support	[17]
S3	L-carnitine (Stage 3)	Metabolic maintenance; consolidation of new adipose microenvironment	Consolidation of reorganized dWAT; maintenance of adipocyte mitochondrial function; stabilization of achieved structural reorganization	[4]
S3	Sorbic acid	Biochemical medium preservation; stabilizer	Maintenance of chemical integrity of active ingredients in the interstitial microenvironment; mild local antimicrobial effect	—
S3	Pyruvic acid (Stage 3)	Senolytic consolidation; regenerative maintenance	Consolidation of residual senescent cell clearance; maintenance of new regenerative microenvironment post-FAS	[14-16]

## Discussion

Patients with CPFP of more than six months’ duration typically show limited functional recovery with currently available standard therapies [1,2]. The HB scale, validated by Ross et al. [8] as a sensitive and reproducible clinical instrument for quantifying facial function, enabled objective tracking of the progression observed in this patient. Our result and its maintenance at nine months without any reinforcement treatments during that period constitute an encouraging finding in the management of these patients. In contrast, botulinum toxin treatment requires periodic injections to sustain synkinesis control and does not restore facial structural support [1,2].

Regarding the bioactive agents used across the different stages, DMAE may act as a motor endplate membrane stabilizer and acetylcholine precursor, potentially optimizing transmission at reinnervated neuromuscular junctions and reducing the ectopic hyperexcitability associated with synkinesis and blepharospasm [9,10], although this mechanism was not directly assessed in the present patient. Unlike botulinum toxin, DMAE is hypothesized to optimize the neuromotor signal rather than suppressing it, which could theoretically preserve residual muscle tone [9,10]. Organic silicon has been described as contributing to extracellular matrix organization and reinforcement of retaining ligaments through collagen cross-linking [17], while acetyl hexapeptide-38 has been reported to promote localized adipogenesis in fat compartments such as the SOOF [11]. Likewise, *C. asiatica* has been described as a neuroprotective and senolytic agent in experimental models, with reported attenuation of the Senescence-Associated Secretory Phenotype (SASP) [12,14,15,18]. Soumyanath et al. demonstrated that *C. asiatica* accelerates peripheral nerve regeneration and stimulates neurite outgrowth [12]; Orhan systematized its neuroprotective potential in experimental models, providing independent support for the use of this active compound in the context of chronic facial denervation [13]. Modulation of the senescent microenvironment via natural senolytics represents a rational and complementary strategy to mechanotransduction [16].

The properties of these active compounds are hypothesized to be additive to the mechanical stimulation of facial adipostructuring, although this combined effect was not directly tested in the present case. Velazco et al. treated 30 patients with the same technique in a separate cohort and documented structural and histological changes in facial tissue, with direct evidence of dWAT reorganization [6], further detailed in the section below. This histological evidence in a population without neuromuscular pathology suggests, but does not confirm, that a similar mechanism could reorganize hemifacial fibrotic dWAT in CPFP. Guntinas-Lichius et al. identified axonal degeneration, loss of trophic support, and fibrosis of the tissue microenvironment as central limiting factors of motor recovery in chronic peripheral paralysis in an animal model [19]; the multimodal treatment described in this report was directed at these three axes, though the relative contribution of each component was not isolated. Our results are consistent with a prior case from our group [20], in which facial adipostructuring was associated with clinical benefit in a patient with Bell’s palsy, raising the hypothesis that this technique may have applicability across different clinical presentations of facial nerve involvement; this remains to be confirmed in larger, controlled studies.

Dermal white adipose tissue as a pathophysiological and therapeutic substrate

Li et al. demonstrated that dWAT can reversibly dedifferentiate into myofibroblasts under TGF-β-mediated pathological stimuli [4]. In chronic CPFP, this process perpetuates hemifacial intradermal fibrosis and blocks local regenerative capacity. Facial adipostructuring, by inducing mechanotransduction in the interseptal compartments [6], may disrupt this degenerative cycle and activate the reservoir of adipose-derived stem cells (ADSCs) present in mature dWAT [18]; this proposed mechanism is supported by preclinical evidence demonstrating that mechanical stimulation of adipose tissue-derived ADSCs activates regenerative signaling pathways via integrin-β1 and cytoskeletal mechanosensors [8], although it was not directly measured in the present case.

Lipolysis induced by mechanotransduction is known to release fatty acids that regulate pro-inflammatory macrophage infiltration [4], a mechanism that may be consistent with the rapid resolution of pain observed from the first session in our patient, though a causal relationship cannot be established from a single case. Velazco et al. provided direct histological evidence of these effects on facial dWAT in a separate cohort: in 100% of post-treatment biopsies, an increase in adipocytes/mm² with smaller diameter and improved organization was documented, along with thickening of interseptal connective tissue septa, greater collagen fiber density, and keratinocyte proliferation [6]. Notably, the same active ingredients applied as mesotherapy without cannula-based mechanical stimulation did not produce equivalent results in that cohort [7], suggesting that mechanotransduction may be an important contributor to the structural changes observed; however, this hypothesis was not independently tested in the present patient. Regarding safety, Velazco et al. reported no serious complications in 103 patients treated with the same technique over a seven-year period, with adverse effects limited to transient erythema (100%), mild edema (46.6%), and bruising (64%), all self-resolving; no infections or severe pain were reported [6]. This is consistent with the tolerability observed in our patient, who experienced only mild transient erythema and edema resolving within 48 hours, with no complications throughout the nine-month follow-up.

Limitations and future recommendations

This report is limited by its single-case design: absence of a control group to establish causality, inability to isolate the individual effect of each treatment component, and absence of electromyographic and/or electroneurographic evaluation that would have allowed objective quantification of nerve conduction recovery. As the patient’s consultation was originally motivated by an aesthetic request rather than a therapeutic intent for facial paralysis, no formal neurological examination, imaging, or standardized baseline functional assessments beyond the HB classification were performed at our clinic; this case report instead emerged from an incidental observation of functional improvement. Future studies prospectively designed to treat CPFP should incorporate complete neurological evaluation, imaging exclusion of alternative etiologies, and standardized baseline functional assessments. Reference to a prior case from the same group [20] represents a limitation of bibliographic independence that future studies with greater author diversity should address. Our work cannot be considered conclusive; prospective controlled studies with larger sample sizes, validated standardized instruments such as the eFACE scale [21] and the Synkinesis Assessment Questionnaire (SAQ) [22], and ideally serial histological and electromyographic evaluation are required to validate these results at a population level.

## Conclusions

This case report describes substantial clinical improvement from HB grade IV to grade I, sustained over nine months, in a patient with CPFP treated with facial adipostructuring and multicomponent biochemical modulation. This finding represents a preliminary observation that suggests potential benefit and generates hypotheses for future investigation, rather than evidence of efficacy or a demonstrated mechanism of action. The proposed pathophysiological framework, dWAT mechanotransduction as a potential disruptor of the fibrosis-denervation cycle, combined with hypothesized cholinergic neuromuscular and senolytic modulation, remains speculative and was not directly measured in this patient. In the absence of a control group, validated functional outcome measures, and neurophysiological assessment, no causal inference regarding treatment efficacy or mechanism can be drawn from this case. Future studies should prioritize prospective controlled designs with electromyographic evaluation, serial histological analysis, and validated instruments such as the eFACE scale and the SAQ. Multicenter trials including populations with different etiologies of peripheral facial paralysis, not limited to Bell’s palsy, would be required to determine the reproducibility, safety, and comparative efficacy of this approach across clinical contexts.
